# Major Honey Bee Diseases and Possibilities to Control Them with Essential Oils

**DOI:** 10.3390/insects17060634

**Published:** 2026-06-16

**Authors:** Yordan V. Hristov, Koycho Koev, Tsvetan Tsvetanov, Ralitsa Balkanska

**Affiliations:** 1 Department “Special Branches”, Institute of Animal Science, Kostinbrod, Agricultural Academy, 1113 Sofia, Bulgaria; yvyh@lycos.com (Y.V.H.); tsvetan28@abv.bg (T.T.); r.balkanska@gmail.com (R.B.); 2 Department of Veterinary Microbiology, Infectious and Parasitic Diseases, Faculty of Veterinary Medicine, Trakia University, 6000 Stara Zagora, Bulgaria

**Keywords:** *Apis mellifera*, honeybee diseases, essential oils, American foulbrood, European foulbrood, *Varroa destructor*, nosemosis, chalkbrood, bee health

## Abstract

Honeybee colonies are affected by bacterial, fungal, viral, microsporidian, and parasitic diseases that impair brood viability, adult survival, productivity, and beekeeping sustainability. Conventional control relies on sanitary measures, antibiotics where permitted, and synthetic acaricides, but these approaches are constrained by residues, resistance, regulation, and variable field performance. This narrative review critically evaluates essential oils as alternative or complementary tools against American foulbrood, European foulbrood, varroosis, nosemosis, chalkbrood, and mite-associated viral pressure. Evidence indicates that thymol, carvacrol, eugenol, cinnamaldehyde, and related constituents can show marked antibacterial, antifungal, acaricidal, antioxidant, and host-supportive activity under controlled conditions. However, most bacterial, fungal, and nosemosis data remain laboratory-based, and direct antiviral applications against honeybee viruses are not field validated. The strongest practical evidence concerns thymol-based control of *Varroa destructor*, where efficacy depends on formulation, release rate, temperature, colony status, and safety margins. Essential oils should therefore be considered components of integrated honeybee health management, not substitutes for established measures. Future studies should prioritize chemical standardization, dose–response testing, colony-level trials, brood and queen safety, sublethal behavior and physiology, gut microbiota, residues, and reproducible delivery systems.

## Simple Summary

Honeybee diseases can weaken colonies, reduce honeybee survival, lower productivity, and threaten pollination, which is important for agriculture and biodiversity. This review summarizes current knowledge on the possible use of essential oils as natural supportive tools for managing important honeybee diseases. The reviewed studies show that some essential oils and their plant compounds can limit the growth of disease-causing organisms or affect parasites under laboratory conditions. The strongest practical evidence is available for thymol-based products against the mite *Varroa destructor*, while evidence for bacterial, fungal, and intestinal diseases is less consistent. The effectiveness of essential oils in real colonies depends on their composition, dose, method of application, temperature, colony condition, and safety for bees and brood. Essential oils should therefore be considered promising complementary tools rather than complete replacements for established disease-control methods. These findings may support more sustainable beekeeping and help protect honeybee colonies that are essential for food production and natural ecosystems.

## 1. Introduction

Honeybees (*Apis mellifera*) are among the most economically and ecologically important pollinators, providing essential pollination services for both agricultural crops and wild plant species [1]. Recent global evidence further shows that insufficient pollinator visitation limits yield in a substantial proportion of crop systems, reinforcing the current agricultural relevance of protecting pollination services [2]. In recent decades, beekeepers in Europe, North America, and other regions have reported increasing colony weakening and elevated colony losses [3,4]. These losses are widely recognized as multifactorial, arising from complex interactions among pathogens, parasites, pesticide exposure, nutritional stress, climate-related factors, and beekeeping practices, rather than from a single causative agent [5]. Among these drivers, infectious and parasitic diseases play a central role in colony decline and reduced productivity.

Honeybee colonies are affected by a broad spectrum of pathogens and parasites, including bacteria, fungi, viruses, microsporidia, and mites [6]. Among bacterial diseases, American foulbrood (AFB), caused by *Paenibacillus larvae* (*P. larvae*), is one of the most destructive brood diseases due to its high lethality and the long-term persistence of spores in hive materials [7,8,9]. A recent review continues to describe AFB as a persistent and internationally relevant brood disease because spore durability, bacterial virulence, diagnosis, and control measures remain central to outbreak management [10]. European foulbrood (EFB), primarily associated with *Melissococcus plutonius* (*M. plutonius*), is another economically significant brood disease, mainly affecting uncapped larvae [11,12]. Updated EFB research standards also emphasize that *M. plutonius* pathogenesis remains incompletely resolved and that modern molecular and diagnostic methods are needed for comparable disease studies [13]. In addition, the ectoparasitic mite *Varroa destructor (V. destructor)* represents one of the most serious global threats to apiculture, owing to both its direct parasitic effects and its role in the transmission and amplification of honeybee viruses, particularly deformed wing virus (DWV) [14,15]. Recent scoping evidence reinforces this assessment by synthesizing *V. destructor* effects on reproduction, immunity, longevity, virus transmission, and the limitations of available control methods [16]. The role of *V. destructor* in viral transmission and amplification is further supported by DWV-specific and global honeybee viral landscape studies [17,18]. Other viral pathogens, including acute bee paralysis virus (ABPV) and chronic bee paralysis virus (CBPV), are also relevant because overt infections can be associated with paralysis, adult mortality, and colony-level weakening [19,20,21].

Microsporidian infections caused by *Nosema apis* (*N. apis*) and *Nosema ceranae* (*N. ceranae*) further contribute to colony health impairment by reducing adult bee lifespan, weakening physiological condition, and lowering colony productivity [22,23]. Chalkbrood, caused by the fungus *Ascosphaera apis* (*A. apis*), is generally less severe than AFB or uncontrolled varroosis but can still weaken colonies and reduce productivity under favorable environmental and colony conditions [24]. These diseases pose a significant challenge to sustainable beekeeping and necessitate effective, safe, and practical control strategies.

Current disease-control options remain limited. The use of antibiotics against bacterial brood diseases is restricted in many countries and may suppress clinical symptoms without eliminating persistent infectious stages [25]. Antimicrobial resistance and residues in bee products raise important regulatory and food safety concerns [26]. Synthetic acaricides are widely applied against *V. destructor*, but their long-term effectiveness is increasingly compromised by resistance development and the accumulation of chemical residues in hive matrices [27,28]. Fumagillin has been used for the control of nosemosis in some regions; however, its application is restricted in Europe due to toxicological and residue-related concerns [29]. For chalkbrood, no consistently effective and widely accepted chemical treatment has been established [30].

These limitations have stimulated growing interest in alternative and complementary approaches for the management of honeybee diseases. Among these, essential oils have attracted particular attention because they contain biologically active compounds with antibacterial, antifungal, acaricidal, antioxidant, and host-supportive properties. The available evidence remains uneven: while numerous studies demonstrate strong in vitro activity, relatively few provide robust data on field efficacy, bee safety, standardized formulations, and practical application under colony conditions. This discrepancy between laboratory results and field applicability represents a major challenge in evaluating essential oils as disease-control agents. Therefore, the present review aims to summarize the major honeybee diseases and to critically assess the potential, mechanisms, limitations, and future perspectives of essential oils in their control.

Within this scope, the review synthesizes evidence on essential-oil chemistry, biological mechanisms, disease-specific efficacy, safety, formulation, and translational limitations relevant to honeybee disease control.

### Review Framework and Literature Selection

This article was prepared as a narrative review using a structured literature-selection framework. The search covered peer-reviewed and otherwise verifiable scientific literature published up to June 2026. PubMed, Scopus, and Web of Science were used as the primary bibliographic databases, and the reference lists of eligible primary studies and recent reviews were screened to identify additional relevant publications. The database search identified 291 records before selection (PubMed, n = 111; Scopus, n = 80; Web of Science, n = 100). The evidence was synthesized qualitatively by disease category, experimental model, biological outcome, safety endpoint, and relevance to practical apicultural application.

Searches were conducted in title, abstract, and keyword fields when supported by the respective database platform. The principal search string combined host, intervention, disease, and outcome terms as follows: (*Apis mellifera* OR honeybee OR “honey bee”) AND (“essential oil” OR “essential oils” OR “plant extract” OR “plant-derived compound” OR “natural product” OR phytochemical OR thymol OR carvacrol OR eugenol OR linalool OR menthol OR eucalyptol OR nanoemulsion OR microencapsulation OR “controlled release”) AND (“American foulbrood” OR “European foulbrood” OR *Paenibacillus larvae* OR *Melissococcus plutonius* OR *Varroa destructor* OR varroosis OR *Nosema apis* OR *Nosema ceranae* OR nosemosis OR *Ascosphaera apis* OR chalkbrood OR “deformed wing virus” OR “acute bee paralysis virus” OR “chronic bee paralysis virus”) AND (“antibacterial activity” OR “antifungal activity” OR “acaricidal activity” OR “antiviral relevance” OR “antioxidant activity” OR toxicity OR “brood safety” OR “queen safety” OR “gut microbiota” OR “field efficacy”).

Disease-specific searches used the same host and intervention blocks combined with targeted pathogen and outcome combinations for American foulbrood and European foulbrood, varroosis, nosemosis, chalkbrood, honeybee viruses, essential-oil formulations, microbiota effects, and colony-level safety. Search terms were adapted to the syntax of each database without changing the underlying concept blocks.

Sources were eligible when they addressed at least one major honeybee disease caused by bacteria, fungi, microsporidia, mites, or viruses; the biological activity of essential oils, plant extracts, formulations, or isolated plant-derived compounds against honeybee pathogens or parasites; mechanisms of antibacterial, antifungal, acaricidal, antiviral, antioxidant, or host-supporting action; adult-bee, brood, queen, colony, or microbiota safety; or semi-field and field application in apiculture. Peer-reviewed primary studies were prioritized for efficacy and safety claims, while review articles, methodological papers, and essential-oil chemistry studies were used for background, interpretation, and citation tracking.

Sources were excluded when bibliographic metadata could not be verified, when the report was not directly relevant to honeybee diseases or essential-oil-based disease control, when the biological outcome was not extractable, when the same data were available in a more complete publication, or when claims could not be traced to peer-reviewed or otherwise verifiable scientific evidence. Studies based only on non-honeybee pathogens, non-apicultural systems, or non-essential-oil interventions were used only when they clarified general mechanisms and were not treated as direct evidence of honeybee-disease control.

Studies presented in Tables were selected when they provided an identifiable oil, compound, extract, or formulation; a defined target disease, pathogen, or parasite; a dose, concentration, or exposure unit; an experimental model and control or comparator information where available; and a measurable efficacy, toxicity, safety, or colony-level endpoint. Priority was given to studies that supported cross-disease comparison, dose–response interpretation, chemical or formulation relevance, field translation, or critical evaluation of evidence strength. Studies with incomplete control information, heterogeneous units, or limited colony-level endpoints were retained only when their limitations could be explicitly interpreted.

The selection pathway consisted of database and citation-chain identification, duplicate and relevance screening, title and abstract assessment against the review scope, full-text or metadata verification where available, and qualitative extraction of the target disease, oil or compound, model, dose, outcome, safety endpoint, and main limitation. Of the 291 records initially identified, 177 were not retained after duplicate checking, relevance screening, bibliographic verification, and citation-chain evaluation. The remaining 114 sources were retained for qualitative synthesis. Evidence strength was interpreted according to experimental scale (in vitro, cage, semi-field, or field), reproducibility, clarity of controls or comparators, dose reporting, bee-safety endpoints, and translational relevance.

## 2. Essential Oils: Definition and Chemical Composition

Essential oils are volatile, lipophilic mixtures of plant secondary metabolites, typically obtained by steam or hydrodistillation from aromatic plants or specific plant parts. According to the Association Française de Normalisation, an essential oil is defined as a product obtained from plant material by distillation with water or steam, by mechanical processing of the epicarp of *Citrus* fruits, or by dry distillation [31]. These oils are generally composed of low-molecular-weight compounds that are poorly soluble in water but readily soluble in organic solvents [32,33,34]. The biological activity of essential oils is closely linked to their chemical complexity. Most essential oils contain dozens of individual constituents, although two or three major compounds often account for a substantial proportion of the total composition [35]. Importantly, minor constituents—even at low concentrations—may significantly influence overall biological activity through additive, synergistic, or antagonistic interactions with dominant compounds. This aspect is particularly relevant in the context of honeybee disease control, where antimicrobial, antifungal, acaricidal, or antioxidant effects may depend on the full chemical profile rather than on a single active component. The chemical composition of essential oils is highly variable and influenced by multiple factors, including plant species, chemotype, geographical origin, climatic conditions, soil characteristics, phenological stage, harvesting time, plant part used, and extraction method [36]. Such variability directly affects the quality, efficacy, and safety of essential oils. Consequently, studies evaluating their activity against honeybee pathogens or parasites should ideally include detailed chemical characterization, as oils derived from the same botanical species may differ substantially in composition and biological activity. The major constituents of essential oils predominantly belong to the chemical classes of terpenoids and phenylpropanoids. Terpenoids constitute the largest group and include monoterpenes and sesquiterpenes, along with their oxygenated derivatives such as alcohols, aldehydes, ketones, phenols, ethers, esters, and epoxides [33,37]. Examples of biologically active terpenoid compounds relevant to honeybee health include thymol, carvacrol, linalool, menthol, geraniol, eucalyptol, and citral. Phenylpropanoids, although generally present in lower concentrations, include important bioactive compounds such as eugenol and cinnamaldehyde [38]. Due to their chemical diversity, essential oils can exert multiple biological effects through different mechanisms of action. Their constituents may disrupt microbial cell membranes, interfere with fungal cell wall integrity, affect mite nervous systems, modulate oxidative processes, and, in some cases, influence honeybee physiological responses. This multi-component and multi-target mode of action underlies the growing interest in essential oils as alternative or complementary tools for controlling major honeybee diseases. However, it also presents challenges related to standardization, reproducibility, dosage optimization, and safety evaluation. Recent biopesticide literature further shows that essential-oil development is increasingly focused on formulation technology, non-target selectivity, environmental fate, and commercialization constraints, rather than on chemical composition or in vitro bioactivity alone [39].

For readability, the principal chemical groups of essential-oil constituents and their relevance to honeybee-disease research are summarized in Table 1 [33,35,36,37,38].

## 3. Biological Roles and Mechanisms of Action of Essential Oils

### 3.1. Antibacterial Activity

The antibacterial activity of essential oils is primarily associated with their hydrophobic and lipophilic properties. Many essential oil constituents can interact with bacterial cell membranes, disrupt lipid organization, increase membrane permeability, and induce leakage of ions and intracellular components, ultimately impairing bacterial viability [40]. Gram-positive bacteria are generally more susceptible than Gram-negative bacteria due to the absence of an outer membrane barrier; however, susceptibility varies depending on bacterial species, oil composition, and experimental conditions [40]. In addition to membrane disruption, essential oils may interfere with bacterial metabolism and virulence-related processes. For instance, carvacrol and p-cymene have been associated with alterations in protein synthesis in *Escherichia coli* [40], while certain essential oils have been reported to reduce protease activity in *P. larvae*, the causative agent of AFB [41]. Given their complex composition, the antibacterial effects of essential oils often result from additive or synergistic interactions among multiple constituents. This multi-target mode of action may reduce the likelihood of rapid resistance development; however, it should not be interpreted as evidence that resistance cannot occur. For spore-forming pathogens such as *P. larvae*, these antibacterial mechanisms primarily concern metabolically active vegetative cells; dormant endospores are structurally protected and metabolically quiescent. Therefore, growth inhibition or protease inhibition should be distinguished from sporicidal activity, inhibition of spore germination, or reduction in spore contamination in hive matrices; the reviewed essential-oil data do not demonstrate reliable inactivation of dormant *P. larvae* endospores [7,8,9,10,41].

### 3.2. Antiviral Relevance

Essential oils and their constituents have been reported to affect viruses in several non-apicultural experimental systems; however, these data do not establish direct antiviral efficacy against honeybee viruses [42]. Accordingly, the virus-related relevance of essential oils in apiculture should be interpreted cautiously and primarily in relation to bee-specific evidence and indirect effects on mite-associated viral pressure.

Honeybee colonies are affected by several clinically relevant ribonucleic acid (RNA) viruses. Deformed wing virus (DWV) is central because its overt clinical expression and colony-level impact are strongly linked to *V. destructor*-mediated transmission and viral amplification [17,18]. The acute bee paralysis virus–Kashmir bee virus–Israeli acute paralysis virus (ABPV–KBV–IAPV) complex may persist covertly but can become highly virulent, especially in mite-infested colonies [19], whereas chronic bee paralysis virus (CBPV) primarily affects adult bees and has increased in some managed honeybee populations [20,21].

Bee-specific studies do not support treating essential oils as direct antiviral therapies. Short-term dietary exposure to phytochemicals, including thymol, has been associated with reduced DWV levels, but higher doses of thymol and clove oil increased mortality [43]. Thyme-oil-supplemented sucrose syrup reduced DWV abundance together with immune-gene changes, suggesting immunomodulation rather than a proven virucidal effect [44]. Conversely, encapsulated thymol and carvacrol did not significantly improve survivorship in an IAPV cage-bioassay [45], and the effects of garlic oil and oregano oil against DWV were mostly non-significant [46]. Thus, no essential-oil treatment is field-validated as a direct antiviral therapy against DWV, ABPV, CBPV, or other honeybee viruses. The most defensible virus-related role is indirect: effective control of *V. destructor* may reduce vector-mediated viral amplification and mite-associated viral pressure, but this should be evaluated by simultaneously measuring mite infestation, viral loads, colony outcomes, dose-dependent toxicity, and bee safety.

### 3.3. Antifungal Activity

The antifungal activity of essential oils is commonly linked to their effects on fungal cell membranes, cell walls, and mitochondrial function. Essential oil constituents may disrupt membrane integrity by interacting with ergosterol or by interfering with its biosynthesis [47]. They may impair mitochondrial activity, reduce adenosine triphosphatase (ATPase) function, increase the production of reactive oxygen species, and inhibit key enzymes involved in cell wall synthesis, including β-glucan synthase, sterol reductases, and chitin synthase [47]. These mechanisms are particularly relevant to chalkbrood, caused by *A. apis*, as inhibition of fungal growth or spore development may reduce pathogen pressure in brood.

These mechanistic observations support antifungal potential, but they do not by themselves establish colony-level chalkbrood control.

### 3.4. Acaricidal Activity

Acaricidal activity is of primary importance in the control of *V. destructor.* Essential oils may affect mites through contact, fumigation, or ingestion-related exposure. At the organismal level, toxicity may involve impaired respiration, disruption of cuticular integrity, behavioral alterations, reduced growth, decreased fecundity, and mortality [48].

At the biochemical level, three mechanisms are particularly relevant: inhibition of acetylcholinesterase, interference with octopaminergic signaling, and modulation of γ-aminobutyric acid (GABA)-gated chloride channels [49,50,51]. Effective varroosis control requires selectivity—compounds must be significantly more toxic to mites than to adult bees, larvae, and queens. Evaluation of essential oils for *Varroa* control should include not only mite mortality but also bee safety, brood effects, formulation stability, and performance under field conditions.

### 3.5. Antioxidant Activity

Antioxidant activity represents another important biological property of many essential oils. From a chemical perspective, lipid peroxidation is a radical chain reaction involving reactive intermediates such as alkylperoxyl radicals. Antioxidants can inhibit these processes by scavenging free radicals, interrupting chain propagation, or forming less reactive intermediates [52].

Several essential oil constituents—particularly phenolic compounds such as thymol, carvacrol, and eugenol—are capable of donating hydrogen atoms to peroxyl radicals, thereby forming relatively stable phenoxyl radicals and limiting oxidative chain reactions. Other compounds may act as termination enhancers or participate in redox reactions that generate less reactive species, as described for γ-terpinene [53]. These antioxidant effects may contribute not only to pathogen control but also to the mitigation of oxidative stress in bees.

### 3.6. Stimulating and Health-Promoting Effects on Bee Colonies

In addition to their direct activity against pathogens and parasites, certain essential oils and plant-derived products have been reported to exert beneficial effects on honeybee health and colony performance. For example, lemongrass and spearmint oils have been associated with increased worker lifespan under cage conditions. Similarly, plant extracts or essential oils derived from garlic, Echinacea, Ganoderma, sage, oregano, thyme, basil, and mint have been reported to influence queen oviposition, brood production, intestinal microbial load, and honey yield in selected studies [54,55]. Recent microbiota-oriented evidence moves this issue beyond general intestinal-load measurements: Ewert et al. [55] evaluated ingested essential oils and propolis extracts in relation to life span, nutrient assimilation, xenobiotic-detoxification markers, and gut bacterial abundance, indicating that supportive products may have microbiota- and physiology-dependent effects rather than uniformly beneficial outcomes. Recent survey-based evidence from Bulgaria adds a field-level context to this interpretation. Koev et al. [56] analyzed climatic conditions during 2021–2024 and a 2024 survey of 70 beekeepers managing 8935 colonies; reported winter mortality in the 2023/2024 season was 2.22% within the surveyed sample and occurred in apiaries where parasite control and herbal complementary feeding schemes were commonly used, but the observational design, self-reported data, and absence of unsupplemented controls preclude causal inference. Although these findings are promising, they should be interpreted with caution. Observed effects may depend on colony condition, season, dosage, route of administration, and formulation. General colony stimulation does not necessarily equate to effective disease control, and further controlled field studies are required to clarify these relationships. Table 2 consolidates these biological activities by target, mechanism, relevance, and representative compounds.

## 4. Efficacy of Essential Oils Against Major Honeybee Diseases

The strength of evidence differs substantially across experimental models. In vitro assays are useful for initial screening of antimicrobial, antifungal, acaricidal, or antiviral activity under controlled exposure conditions, but they do not reproduce colony architecture, brood thermoregulation, social immunity, food distribution, volatilization dynamics, or pathogen transmission within the hive. Cage assays provide information on individual-level survival, toxicity, infection intensity, and dose response, yet they exclude queen status, brood effects, colony demography, foraging behavior, and environmental variation. Semi-field studies offer an intermediate level of biological relevance by testing delivery under apiary conditions, whereas field trials provide the strongest practical evidence because they assess colony-level efficacy, safety, treatment delivery, seasonality, and recurrence. Therefore, laboratory findings should be interpreted as hypothesis-generating unless they are supported by reproducible semi-field or field data with appropriate controls, standardized outcome measures, and concurrent assessment of bee and brood safety. A balanced interpretation also requires explicit consideration of negative and inconclusive results, because selective emphasis on inhibitory activity or mite mortality may overestimate practical value when unsuccessful field applications, dose-limiting toxicity, weak replication, or formulation failure are not given equal weight.

### 4.1. American Foulbrood (AFB)

AFB, caused by *P. larvae*, is among the most extensively studied honeybee diseases in the context of essential oils. The available evidence is strongest at the in vitro level, where experimental conditions—such as oil composition, concentration, and exposure time—are well defined. Calderone et al. [57] reported that cinnamon oil at 10 µg/mL (reported as 10 ppm) inhibited *P. larvae* growth for 72 h, whereas camphor and citronellal oils were active at 100 µg/mL (reported as 100 ppm); bay, clove, and oregano oils, as well as thymol, required 1000 µg/mL (reported as 1000 ppm), and α-terpinene required 10,000 µg/mL (reported as 10,000 ppm) under comparable conditions. Alippi et al. [58] screened eight essential oils and identified lemongrass and thyme oils as the most effective inhibitors of *P. larvae* growth. Subsequent studies have confirmed antibacterial activity for cinnamon oil, cinnamaldehyde, eugenol, thymol, tea tree oil, oregano oil, clove oil, and grapefruit oil [41,59,60,61,62]. These findings indicate that *P. larvae* is susceptible to several phenolic and phenylpropanoid constituents, particularly thymol, carvacrol, eugenol, and cinnamaldehyde. Fuselli [59] and co-workers reported a synergistic inhibitory effect for a mixture composed of 62.5% thyme oil, 12.5% cinnamon oil, and 25% thymol, suggesting that combinations of oils or purified constituents may be more effective than single compounds. This observation highlights a key concept: the biological activity of essential oils typically reflects interactions among multiple constituents rather than the effect of a single dominant compound. Consequently, these in vitro assays should be interpreted primarily as evidence of inhibition of vegetative-cell growth or activity of *P. larvae* under culture conditions; they do not demonstrate endospore inactivation unless spore viability, viable-spore counts, or germination after exposure is directly measured [7,8,9,10,57,58]. Thus, the reviewed in vitro evidence supports antibacterial activity against vegetative *P. larvae* cells, but not sporicidal activity against dormant endospores.

Despite robust in vitro evidence, field and semi-field data for AFB remain limited and inconsistent. Floris et al. [63] administered *Cinnamomum* sp. essential oil at 400 mg/kg in semi-solid feed to colonies affected by AFB and reported a reduction in clinical signs, while also emphasizing challenges related to treatment timing and delivery. Gende et al. [60] applied *Cinnamomum zeylanicum* essential oil at two doses of 1000 µg/mL per hive at 7-day intervals and observed a significant reduction in infected larvae compared with untreated controls. Albo et al. [64] reported that several essential oils (savory, thyme, lemongrass, oregano) and their mixtures failed to prevent or eliminate clinical AFB under field conditions, and Puvača [65] reported insufficient efficacy of lavender oil. These contrasting colony-level outcomes indicate that the predictive value of antibacterial screening is limited when treatment delivery, brood exposure, infection pressure, and persistence of *P. larvae* endospores are not evaluated under realistic hive conditions. The discrepancy is biologically important because transient suppression of vegetative cells does not necessarily reduce the infectious spore reservoir in brood remains, wax, honey, or equipment [8,9,10,63,64,65].

Essential oils cannot currently be considered established treatments for AFB. Rather, they should be viewed as promising candidates for complementary control strategies, requiring further validation at the colony level, particularly with respect to spore persistence and long-term disease dynamics. The current evidence is therefore best classified as strong for in vitro inhibition of vegetative cells, but not as evidence of reliable sporicidal activity against dormant endospores, and weak to moderate for reproducible colony-level disease control. The negative field results reported for savory, thyme, lemongrass, oregano, mixed-oil applications, and lavender oil are therefore not peripheral observations; they define the present boundary between antibacterial potential and dependable disease management under apiary conditions [64,65]. Future AFB studies should therefore report vegetative-cell minimum inhibitory concentration (MIC), inhibition-zone, or virulence-factor endpoints separately from endospore-germination, endospore-viability, contaminated-matrix, and recurrence endpoints.

Recent laboratory evidence has expanded the foulbrood evidence base by directly testing a broad panel of essential oils against both *P. larvae* and *M. plutonius* while also assessing worker-bee tolerance to vapor exposure. Marianelli and Narciso identified oregano, juniper, sage, thyme, cinnamon, cumin, clove, and black pepper oils among the most active against foulbrood bacteria; however, bee tolerance differed among oils and toxicity increased with longer exposure time [66]. This study is valuable because it links pathogen inhibition with adult-bee safety screening, but it remains laboratory-based; larval bioassays and field trials are still required before clinical disease-control claims can be made.

### 4.2. European Foulbrood (EFB)

Compared with AFB, substantially fewer studies have evaluated essential oils against EFB, primarily associated with *M. plutonius*.

Calderone et al. [57] demonstrated that cinnamon oil and thymol at 10 µg/mL (reported as 10 ppm) completely inhibited *Paenibacillus alvei (P. alvei)*, a frequent secondary invader in EFB, over 72 h. Studies using *Polygonum bistorta* essential oil have reported activity against both *P. larvae* and *M. plutonius*, although the inhibitory effect against *M. plutonius* was weaker. Santos et al. [61] reported strong inhibitory activity of tea tree oil, including nanoparticle formulations, against both pathogens, while Hassona [67] suggested potential field relevance of clove oil. Despite these encouraging findings, the evidence base for EFB remains limited. Importantly, EFB pathogenesis is multifactorial and influenced by larval nutrition, colony strength, environmental stressors, and secondary bacterial invaders. Consequently, antibacterial activity against isolated pathogens cannot be directly extrapolated to reliable disease control at the colony level. The limited number of studies directly targeting *M. plutonius*, together with reliance on surrogate or secondary bacterial targets in some experiments, increases uncertainty and limits reproducibility across EFB models. The recent inclusion of *M. plutonius* in direct essential-oil screening strengthens the relevance of EFB-specific evaluation, but it also confirms that antibacterial activity must be interpreted together with exposure-related bee toxicity and colony-level validation [66]. From a practical standpoint, the EFB evidence remains particularly vulnerable to overinterpretation because inhibition of *P. alvei* or isolated *M. plutonius* does not demonstrate reversal of brood disease, prevention of recurrence, or reliable performance in nutritionally stressed colonies.

### 4.3. Varroosis

Varroosis, caused by *V. destructor*, represents the most practically relevant area for the application of essential oils in apiculture. Numerous laboratory studies have demonstrated acaricidal activity of essential oils and their constituents; however, results are often difficult to compare due to methodological heterogeneity, including differences in mite collection, exposure routes, dose expression, environmental conditions, and mortality assessment. Moreover, bee and brood toxicity are not consistently evaluated, despite being critical for practical application. Evidence quality is strongest when studies report mite mortality together with bee mortality, brood effects, exposure route, temperature, release kinetics, and an established acaricide or untreated control. Negative or suboptimal outcomes are especially important in this section because a treatment that reduces mite numbers only partially, or only under narrow temperature and delivery conditions, may still leave colonies exposed to damaging mite and virus pressure.

A wide range of essential oils have demonstrated acaricidal activity against *V. destructor*, including oils from *Tagetes minuta*, *Schinus molle*, *Heterotheca latifolia*, *Heterothalamus alienus*, *Citrus* spp., *Cymbopogon citratus*, eucalyptus, clove, anise, oregano, mint, and thyme [68,69,70,71,72,73]. Quantitative studies provide more meaningful comparisons when endpoint terminology and dose expression are reported consistently. For example, Eguaras et al. [68] reported a median lethal dose (LD_50_; DL_50_ in the original article) of 4.37 mg/cage for *Tagetes minuta* oil after 24 h, while Ruffinengo et al. [69] reported an LD_50_ of 2.65 µL/cage for *Schinus molle* oil, with a selectivity ratio above 16, indicating higher toxicity to mites than to bees. Sabahi et al. [71] reported median lethal concentration (LC_50_) values of 304.9 µg/mL for anethole and 474.13 µg/mL for *Cymbopogon citratus* oil, with relatively low toxicity to bees and larvae. *Citrus* oils also showed substantial mite mortality in vitro, with bergamot, grapefruit, and lemon oils achieving approximately 80%, 70%, and 69% mortality, respectively [72]. These quantitative data highlight both the potential and a major limitation of the field: differences in endpoint type, dose expression, exposure route, and experimental system limit direct comparison and ranking of candidate oils. More recent chemically characterized studies reinforce the need to evaluate acaricidal activity together with bee safety. Erdoğan reported strong fumigant and repellent effects of *Origanum acutidens* and *Satureja hortensis* oils against *V. destructor*, with larval mortality remaining below 5% in the tested treatments [74]. Manzo et al. [75] showed that Patagonian wild-plant oils differed markedly in toxicity to mites, adult bees, and larvae, indicating that selectivity is oil-specific and cannot be assumed from botanical origin alone.

Field efficacy depends strongly on formulation and delivery systems. Laboratory activity does not necessarily translate into effective hive-level control, as factors such as evaporation rate, temperature, colony strength, brood presence, and treatment duration determine actual exposure. Thymol remains the most substantiated essential-oil-based compound for varroosis control. Lindberg et al. [76] reported high mite mortality with thymol and clove oil under laboratory conditions, while Gashout and Guzmán-Novoa [77] reported LC_50_ values of 56.1 µg/vial for thymol and origanum oil in a glass-vial residual bioassay. Begna et al. [78] reported LC_50_ values of 71 µg/mL for thymol and 42 µg/mL for a thymol–carvacrol mixture, with the mixture showing higher toxicity to mites than fluvalinate in that experimental model. Consequently, thymol-based products occupy a different evidentiary category from many other essential oils: their practical relevance is supported by formulation-dependent field evidence, whereas most non-thymol oils remain supported mainly by laboratory or small-scale assays. 

Recent studies from 2024 to 2026 make this formulation-dependent evidence more explicit. Semi-field and field testing of *Citrus bergamia* and *Citrus limon* oils showed no bee toxicity in semi-field cages, but field mite mortality at the highest concentrations reached only 40.3% and 50.7%, respectively, supporting the need for gradual and controlled release under hive conditions [79]. In contrast, colony-level testing of peppermint, thyme, and eucalyptus nanoemulsions reported concentration-dependent reductions in *Varroa* infestation, with the highest efficacy for thyme nanoemulsion at 200 µg/mL (reported as 200 ppm), although the authors emphasized that brood safety, residues, and broader bee-health endpoints still require evaluation [80]. A thymol nanoemulsion colony trial similarly suggested potential dose reduction, but the highest numerical mite fall did not translate into statistically clear superiority among groups, showing that nanoformulation alone does not guarantee robust colony-level efficacy [81]. Integrated pest-management studies also remain important: a field trial combining registered amitraz and thymol products achieved rapid *Varroa* control comparable to amitraz emulsifiable concentrate at 21 days, but lower bee populations in the combination group indicated that timing, dose, and colony-strength effects require optimization [82]. These examples show that recent studies do not uniformly support immediate practical adoption: moderate field mite mortality, statistically unclear treatment separation, and reduced bee populations in some combination-treatment groups all argue for cautious interpretation rather than simple classification as successful alternatives [79,81,82]. To provide a more detailed comparison of acaricidal efficacy, selectivity, and formulation-dependent outcomes, representative experimental data on essential oils tested against *V. destructor* are summarized in Table 3.

Delivery method appears to be a critical determinant of efficacy. Sabahi et al. [87] reported 57.8% efficacy for oregano oil delivered via impregnated absorbent pads, whereas efficacy increased to 97.4% when continuous-release vaporizers were used. These findings clearly demonstrate that formulation and delivery systems are not merely technical details but key determinants of biological and practical outcomes. The lower efficacy of impregnated pads compared with continuous-release vaporizers illustrates how a biologically active oil can fail operationally when release kinetics do not sustain an effective hive concentration [87].

### 4.4. Nosemosis

Nosemosis caused by *N. apis* and *N. ceranae* cannot be evaluated only by final spore counts because *Nosema* spp. are obligate intracellular microsporidia with a stage-dependent infection cycle. After ingestion, mature spores germinate in the honeybee midgut and extrude a polar tube, through which the sporoplasm is transferred into epithelial cells; subsequent merogony and sporogony generate new spores and contribute to epithelial dysfunction, altered cell turnover, and spore shedding [22,23,89,90]. This biology determines which mechanisms should be considered when interpreting essential-oil studies: inhibition of spore germination or polar-tube extrusion would affect invasion, suppression of intracellular replication would reduce parasite multiplication after cell entry, and stimulation of epithelial renewal would reflect a host-mediated recovery process rather than direct parasite killing.

Against this framework, thymol studies provide outcome-level anti-*Nosema* evidence but do not yet define the affected parasite stage. Maistrello et al. [91] reported reduced infection levels in bees fed thymol-supplemented candy, Costa et al. [92] found reduced *N. ceranae* development and improved longevity after thymol administration, and Glavinić et al. [93] observed lower spore loads by day 15 post-infection together with changes in oxidative-stress and immune-related parameters. These findings support biological activity, but their endpoints mainly combine parasite burden, host survival, and host physiological responses; they do not directly show whether thymol blocks polar-tube extrusion, acts on intracellular meronts or sporonts, or indirectly favors renewal of damaged midgut epithelium.

The same interpretive limitation applies to other plant-derived products. Extracts from *Laurus nobilis*, *Aster scaber*, *Artemisia dubia*, *Aristotelia chilensis*, *Ugni molinae*, *Eleutherococcus senticosus*, and *Andrographis paniculata* have been associated with reduced spore development, lower infection intensity, or improved bee survival in experimental studies [94]. However, these endpoints remain biologically downstream unless paired with controlled spore-germination assays, polar-tube extrusion measurements, intracellular parasite quantification, and histopathological assessment of the midgut epithelium. Thus, the current plant-extract evidence broadens the candidate pool but does not yet resolve whether activity is directed at the spore, the intracellular parasite, or the host tissue response.

Field evidence should be interpreted even more cautiously. Özüiçli et al. [95] evaluated thyme, peppermint, and eucalyptus essential oils under field conditions and reported the highest efficacy for thyme oil (84%), followed by peppermint (77.45%) and eucalyptus (76.10%). At colony level, these values indicate possible practical relevance, but they remain downstream outcomes that may be influenced by initial infection intensity, feeding route, season, colony demography, nutrition, gut microbiota, queen and brood status, and diagnostic timing. Future anti-*Nosema* studies should therefore link spore-load reduction with polar-tube extrusion, intracellular parasite burden, midgut histopathology, epithelial-renewal markers, survival, food intake, microbiota effects, and colony-level recovery before assigning a precise mode of action or recommending essential oils as field-ready nosemosis treatments.

### 4.5. Chalkbrood

Chalkbrood, caused by *A. apis*, has been extensively investigated in in vitro studies. Calderone et al. [57] reported that camphor and citronellal oils at 100 µg/mL (reported as 100 ppm) inhibited fungal growth for 72 h, while cinnamon oil at the same concentration maintained inhibition for up to 168 h. Larrán et al. [96] found that coriander oil exhibited antifungal activity at concentrations of 700–900 µL/L, whereas basil and *Tagetes minuta* oils were effective at 800 µL/L. Additional studies have identified antifungal activity in essential oils derived from thyme, cinnamon, clove, pelargonium, *Armoracia rusticana*, *Origanum vulgare*, *Cymbopogon* spp., *Allium sativum*, *Litsea cubeba*, and *Cinnamomum zeylanicum* [97,98,99,100]. Pusceddu et al. [100] reported minimum fungicidal and sporicidal concentrations in the range of 200–400 µg/mL (reported as 200–400 ppm) for oils from *Thymus herba-barona*, *Thymus capitatus*, and *Cinnamomum zeylanicum*. As with other honeybee diseases, field validation remains limited. Mourad et al. [101] evaluated cedar oil and thymol following laboratory confirmation of antifungal activity and reported a 100% reduction in chalkbrood mummies with 4% cedar oil, while thymol achieved reductions ranging from 63.22% to 96.94%. These findings are particularly valuable because they link laboratory antifungal effects with colony-level outcomes. However, the evidence remains vulnerable to overinterpretation when studies rely on agar-based inhibition zones or short-term fungistatic endpoints without assessing brood-level disease recurrence, hygienic behavior, or environmental drivers. The high reduction reported in a single field context should therefore not obscure the possibility of seasonal recurrence, colony-to-colony variability, or failure under different brood-temperature and humidity conditions [101].

Chalkbrood development is strongly influenced by environmental and colony-level factors, including brood temperature, humidity, colony strength, and hygienic behavior. Therefore, antifungal activity observed under laboratory conditions should be interpreted as preliminary unless supported by robust field data. Replication across seasons and apiary contexts is particularly important because colony strength and brood microclimate can modify disease expression independently of antifungal activity.

**Figure 1:**
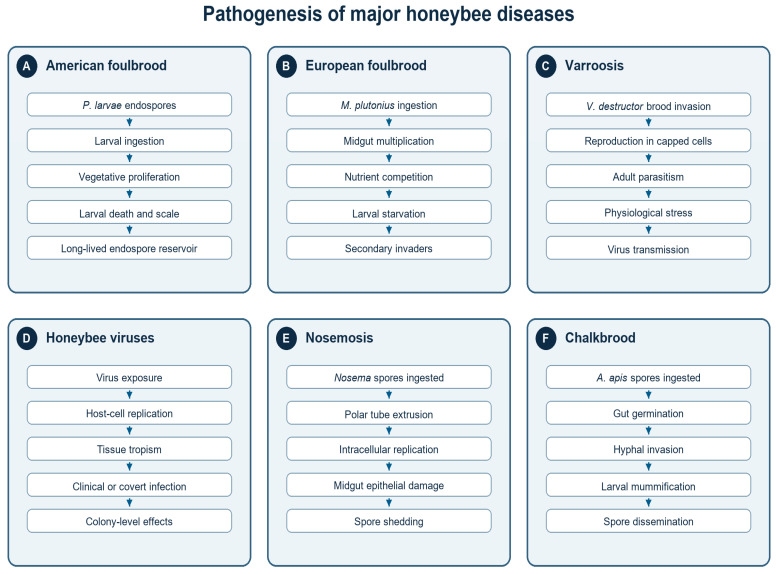
Pathogenesis-oriented framework for interpreting essential-oil evidence across major honeybee diseases. The panels summarize the principal infection, parasitism, or viral-disease sequences for AFB, EFB, varroosis, honeybee viral infections, nosemosis, and chalkbrood.

The main pathogenesis pathways relevant to interpreting disease-specific efficacy and evidence translation are summarized in Figure 1.

To facilitate a structured comparison of experimental designs, control or comparator information, doses, biological outcomes, and model-level limitations, selected representative studies are reorganized by disease group and experimental scale in Table 4.

To distinguish experimental promise from practical applicability, the available evidence is summarized according to the dominant experimental model, relative strength of support, and major limitations affecting reproducibility, bias, and translation to beekeeping practice (Table 5).

## 5. Limitations and Future Perspectives

Despite the growing body of literature on essential oils, several key limitations still constrain their practical application in beekeeping.

A major limitation is the chemical variability of essential oils. Their composition depends on plant species, chemotype, geographical origin, phenological stage, extraction method, and storage conditions. In the absence of detailed chemical characterization, it remains difficult to compare results across studies or to reproduce reported efficacy.

Another important limitation is methodological heterogeneity. Studies differ substantially in pathogen strains, mite collection procedures, exposure routes, concentration units, incubation conditions, treatment duration, and outcome measures. This issue is particularly evident in studies on *V. destructor*, where contact toxicity, fumigation, direct application, and field delivery systems may yield markedly different results. The development of standardized experimental protocols is therefore essential to distinguish broadly applicable findings from laboratory-specific observations. Evidence quality is further constrained when studies use small or unspecified sample sizes, lack untreated or reference-treated controls, do not randomize colonies or cages, omit blinding of outcome assessment, or report only positive endpoints.

Bee safety also remains a critical concern and should be interpreted beyond acute mortality. Essential oils are biologically active substances and may cause toxicity, repellency, feeding disruption, brood effects, queen impairment, or microbiota-mediated adverse effects when used at inappropriate doses. Accordingly, efficacy against pathogens or parasites must always be evaluated alongside organismal and microbiome-level safety at the level of adult bees, larvae, queens, brood, and entire colonies. The honeybee gut microbiota contributes to nutrient metabolism, immune maturation, and colonization resistance, and disruption of core symbionts can increase susceptibility to opportunistic pathogens [102,103]. Because ingested essential oils and propolis extracts can alter gut bacterial abundance and host physiological markers [55], broad-spectrum antimicrobial activity should be regarded as a potential safety liability as well as a therapeutic property. Unselective suppression of beneficial taxa, including *Snodgrassella*, *Gilliamella*, Lactobacillus-related clades, and *Bifidobacterium*, could weaken gut barrier functions, digestive resilience, and natural resistance to secondary infections or dysbiosis-associated stress.

Recent risk-assessment work with an essential-oil mixture and isolated constituents found limited acute lethal toxicity under the tested laboratory conditions but compound-specific sublethal biochemical and gene-expression responses, reinforcing that safety assessment should include enzyme activity, detoxification pathways, feeding exposure, contact exposure, gut-microbiota composition, pathogen-challenge susceptibility, and chronic endpoints rather than mortality alone [104].

Formulation represents an additional challenge. High volatility and chemical instability may reduce field efficacy or result in excessive peak exposure. Advanced delivery systems, such as controlled-release formulations, microencapsulation, nanoemulsions, and optimized carriers, offer potential solutions but require thorough validation under realistic colony conditions. Recent nanoemulsion and controlled-release studies therefore support a formulation-centered research direction, but they also show that carriers, surfactants, release kinetics, repeated dosing, residue behavior, brood safety, and seasonality must be validated before nanoemulsions, microencapsulation, or other advanced delivery systems can be recommended for routine apicultural use [79,80,81,104]. A broader recent review of controlled-release essential oil formulations further supports this caution by identifying microcapsules, mesoporous silica, polymeric nanoparticles, Pickering emulsions, and metal–organic framework carriers as strategies for stabilizing volatile compounds and controlling release, while emphasizing scalability, environmental fate, and regulatory acceptance as unresolved barriers to practical deployment [105].

Within this broader safety and formulation context, exposure assessment should specify the route and duration of contact, fumigation, ingestion, and matrix-associated residues in wax, honey, pollen stores, brood food, and other hive materials. These variables determine whether a formulation produces transient worker exposure, sustained brood exposure, or colony-wide vapor exposure, and they should be reported together with chemical composition, release kinetics, dose, temperature, brood status, and colony strength [55].

Brood and queen endpoints are particularly important because adult-worker tolerance does not guarantee colony safety. In vitro larval assays showed lethal and sublethal effects of thymol in *Apis mellifera* larvae, demonstrating that larval exposure through contaminated food can be safety-limiting [106]. Field studies of thymol formulations further show that efficacy, persistence, residues, and colony tolerance must be evaluated together [107], and botanical-oil spray experiments reported substantial queen-loss concerns under some treatments [108]. Direct queen-specific toxicity studies remain scarce; therefore, queen mortality, egg laying, retinue behavior, supersedure, brood pattern, and worker acceptance should be mandatory endpoints before an essential-oil formulation is considered colony safe.

Sublethal effects can occur without immediate mortality. Thymol exposure has been associated with concentration-dependent genotoxic effects in a honeybee cell line [109], age-dependent avoidance and fanning responses to Apiguard [110], altered olfactory memory and expression of neural target genes [111], changes in olfactory-gustatory learning congruency [112], and modified hygienic and brood-removal behavior [113]. These findings show that safety cannot be inferred from acute mortality alone, because volatile oils may affect sensory processing, disease-related social behavior, ventilation, feeding-related responses, and colony hygiene at sublethal doses.

Colony organization also depends on pheromone-mediated regulation. Queen mandibular, brood, alarm, Nasonov, tergal, Dufour, and tarsal gland pheromones coordinate queen-worker interactions, retinue behavior, brood care, defense, orientation, and division of labor [114]. Because essential oils are strong volatile odorants and thymol studies show effects on olfactory memory, sensory integration, and behavior [110,111,112,113], formulations should be tested for possible interference with pheromone perception and colony communication. At present, direct colony-level evidence that essential oils disrupt honeybee pheromone systems remains limited; this should be treated as an important safety knowledge gap rather than as an established field effect.

The translation of laboratory findings into beekeeping practice remains limited. Many studies demonstrate promising antimicrobial, antifungal, acaricidal, or antiparasitic activity under controlled conditions, but fewer studies assess long-term colony-level outcomes, seasonal variation, interactions with other management practices, residue risks, or performance under commercial apiary conditions. Contradictory findings should therefore be interpreted in relation to experimental scale, dose expression, formulation, delivery method, colony condition, pathogen pressure, and the biological endpoint measured rather than treated as simple confirmation or rejection of a given oil.

Commercial implementation presents an additional set of limitations. Essential-oil-based products require reproducible botanical sourcing, chemical standardization, batch-to-batch quality control, stability during storage, predictable release under variable hive temperatures, acceptable residues in honey and wax, clear withdrawal or use instructions, beekeeper-safe handling, and regulatory authorization. These requirements are difficult to satisfy when active constituents are volatile, chemically variable, or effective only within a narrow dose window. Consequently, a formulation may be scientifically promising but commercially unsuitable if it cannot deliver consistent efficacy, safety, shelf life, and compliance at scale [104,105].

Future research should prioritize the use of chemically characterized oils, well-defined dose–response studies, standardized laboratory methodologies, comprehensive safety assessments, controlled field trials, and formulation development. Importantly, essential oils should be evaluated as components of integrated disease management strategies rather than as universal substitutes for established sanitary, veterinary, or acaricidal measures. To summarize the key constraints identified in the current literature and highlight priorities for future research, the main limitations and recommended directions for essential-oil-based honeybee disease control are presented in Table 6. Recent work also indicates that future studies should include microbiota-sensitive endpoints [55], together with sublethal biochemical markers and compatibility with integrated pest-management schedules, especially when essential oils are combined with established acaricides or delivered through novel formulations [82,104]. Climate-adapted field studies should also separate the effects of parasite control, nutrition, and herbal supplementation, because recent survey data suggest associations with winter survival but do not establish causal efficacy of specific plant-derived products [56]. Publication of neutral or negative findings should be encouraged, because failed field applications, dose-limiting toxicity, weak selectivity, and commercially impractical formulations are essential for defining realistic use conditions.

## 6. Conclusions

Essential oils exhibit considerable biological potential for the control of major honeybee diseases. Their antibacterial, antifungal, acaricidal, antioxidant, colony-supportive, and virus-related properties provide a strong scientific basis for their evaluation as alternative or complementary tools in apiculture. Among these applications, the most robust practical evidence supports the use of thymol and thymol-based formulations for the control of *V. destructor*. For AFB, EFB, nosemosis, and chalkbrood, numerous essential oils demonstrate promising in vitro activity; however, evidence from well-controlled field studies remains limited. Critical gaps persist in relation to chemical standardization, dose optimization, formulation strategies, delivery systems, and comprehensive evaluation of bee and brood safety. At the same time, the evidence does not support a uniformly positive assessment: several oils show weak, inconsistent, or non-transferable performance once tested beyond controlled laboratory conditions. Practical suitability therefore depends on colony-level safety evidence that includes reproductive, developmental, behavioral, microbiota-related, and communication-mediated endpoints.

The practical effectiveness of essential oils depends not only on their intrinsic biological activity but also on multiple interacting factors, including chemical composition, concentration, formulation, method of application, and colony-level conditions. Therefore, essential oils should not be regarded as universal substitutes for conventional disease-control measures. Instead, they should be considered promising components of integrated honeybee health management strategies. The most recent evidence strengthens this conclusion by showing that updated formulations can improve exposure and mite reduction, but also that field efficacy and colony strength remain decisive criteria for translation into practice [79,80,81,82]. A realistic assessment should therefore treat essential oils as candidates requiring rigorous product-level validation, not as ready substitutes for established sanitary, nutritional, and veterinary measures.

## Figures and Tables

**Table 1:** Principal chemical classes of essential-oil constituents relevant to honeybee-disease research.

Chemical Class or Compositional Group	Representative Constituents	Relevance to Honeybee-Disease Research	Main Interpretive Limitation
Monoterpene hydrocarbons	p-Cymene, α-terpinene, γ-terpinene, limonene	Contribute to volatility, membrane interaction, release behavior, and potential synergistic effects within whole oils.	High volatility and chemotype dependence make activity difficult to compare without full chemical profiling.
Oxygenated monoterpenes	Thymol, carvacrol, linalool, menthol, geraniol, eucalyptol, citral	Frequently associated with antibacterial, antifungal, acaricidal, antioxidant, and sensory effects relevant to bee pathogens, mites, and bee safety.	Potency depends on dose, formulation, exposure route, and selectivity between target organisms and bees.
Sesquiterpenes and oxygenated sesquiterpenes	β-Caryophyllene, farnesol, nerolidol and related compounds	May contribute to persistence, odor profile, membrane activity, and whole-oil bioactivity.	Often reported less consistently than dominant monoterpenes, limiting reproducibility across studies.
Phenylpropanoids	Eugenol, cinnamaldehyde	Important contributors to antibacterial and antifungal activity, including effects relevant to brood-disease pathogens.	Strong in vitro activity does not by itself establish colony-level efficacy or bee safety.
Minor constituents and whole-oil matrix	Low-abundance alcohols, aldehydes, ketones, esters, ethers, phenols, and epoxides	Can modify activity through additive, synergistic, or antagonistic interactions with dominant compounds.	Single-compound interpretation may overestimate reproducibility when the full oil profile is not characterized.

**Table 2:** Main biological activities and mechanisms of action of essential oils relevant to honeybee disease control.

Biological Activity	Main Targets	Representative Mechanisms	Relevance	Representative Compounds
Antibacterial	Bacterial membrane, cell wall, metabolic processes	Membrane disruption; increased permeability; leakage of intracellular components; interference with protein synthesis and enzymatic activity	AFB; EFB	Thymol; carvacrol; eugenol; cinnamaldehyde
Antiviral relevance	Honeybee-virus evidence limited; *Varroa destructor*-associated viral pressure	Possible immunomodulation; indirect reduction in vector-mediated viral amplification through mite control	No field-validated direct antiviral therapy; most defensible role is indirect through *V. destructor* control	Thymol; thyme oil; other phytochemicals
Antifungal	Fungal membrane; ergosterol pathways; mitochondria; cell wall	Membrane alteration; interference with ergosterol biosynthesis; mitochondrial dysfunction; inhibition of cell wall synthesis	Chalkbrood	Thymol; citral; eugenol; cinnamaldehyde
Acaricidal	Arthropod nervous system; respiration; cuticle	Acetylcholinesterase inhibition; interference with octopaminergic signaling; modulation of GABA-gated chloride channels	Varroosis	Thymol; menthol; citral; geraniol
Antioxidant	Radical-mediated oxidation; lipid peroxidation	Radical scavenging; hydrogen donation; termination of oxidative chain reactions	Supportive role in bee oxidative stress physiology	Thymol; carvacrol; eugenol; γ-terpinene
Colony-supportive	Adult bee physiology; brood production; gut microbiota	Possible stimulation of feeding behavior; enhancement of brood production; modulation of intestinal microbiota	Potential supportive effects; not equivalent to proven disease control	Lemongrass; spearmint; oregano; thyme

**Table 3:** Selected experimental data on the acaricidal activity of essential oils against *V. destructor*.

Essential Oil/Compound	Study Type	Reported Efficacy/Toxicity Endpoint	Bee Safety/Selectivity	Reference
Thymol	In vitro and field	High mite mortality; LC_50_ values reported in laboratory assays; field efficacy may exceed 90% under optimal conditions	Generally acceptable at controlled doses; temperature and release rate are critical	[76,77,83,84,85,86]
Peppermint, thyme, and eucalyptus nanoemulsions	Colony-level field study	Dose-dependent Varroa reduction; thyme nanoemulsion at 200 µg/mL (reported as 200 ppm) showed the highest reported efficacy	Brood safety, residue profile, and long-term colony endpoints still require validation	[80]
Thymol nanoemulsion	Colony-level field study	Highest numerical mite fall reported at 25 µg/mL (reported as 25 ppm); between-group superiority was not statistically clear	Potential dose reduction, but further seasonal and colony-level safety studies are required	[81]
Thymol + carvacrol	In vitro	LC_50_ ≈ 42 µg/mL in short-exposure assays	Higher toxicity to mites than fluvalinate in the cited study	[78]
*Origanum* spp. (oregano oil)	In vitro and field	High laboratory mite mortality; field efficacy strongly dependent on continuous-release delivery	Dose-dependent; formulation determines safety and efficacy	[73,87]
*Citrus* spp. oils	In vitro	Bergamot, grapefruit, and lemon oils caused ~69–80% mite mortality in laboratory assays	Low bee toxicity reported in selected assays	[72]
Citrus bergamia and Citrus limon	Semi-field and field	Highest field mite mortality reached 40.3% and 50.7%, respectively	No bee toxicity in semi-field cages; controlled-release delivery is needed	[79]
Origanum acutidens, Satureja hortensis, and Juniperus communis	Laboratory gas chromatography-mass spectrometry (GC-MS)-linked fumigation and repellency assays	Strongest LC_50_ values reported for Satureja hortensis and Origanum acutidens oils	Honeybee larval mortality remained below 5% under tested conditions	[74]
Patagonian wild-plant oils	Laboratory mite, adult-bee, and larval assays	Acaricidal activity varied strongly among oils and exposure times	Selectivity differed between mites, adult bees, and larvae	[75]
*Tagetes minuta*	In vitro	LD_50_ ≈ 4.37 mg/cage (24 h; complete-exposure assay)	Acceptable selectivity reported	[68]
*Schinus molle*	In vitro	LD_50_ ≈ 2.65 µL/cage (24 h; complete-exposure assay)	High selectivity ratio reported	[69]
Microencapsulated oils	In vitro/formulation-oriented	Microencapsulated *Acantholippia seriphioides* and *Schinus molle* oils showed promising acaricidal activity	Improved formulation may enhance applicability; field validation required	[70]
*Cymbopogon citratus* and anethole	In vitro	LC_50_ ≈ 474 µg/mL for Cymbopogon citratus oil and 304.9 µg/mL for anethole	Relatively safe for bees and larvae in the cited study	[71]
*Cinnamomum*/eucalyptus formulations	Laboratory and field	Substantial field reduction when application method and release profile are optimized	Delivery system is a key determinant of efficacy and safety	[88]

Note: Concentration units are standardized as µg/mL for ppm and mg/mL values and as µL/mL for µL/L values. LD_50_ values in mg/cage or µL/cage and feed-, hive-, or formulation-level doses are retained as exposure-unit endpoints.

**Table 4:** Selected experimental studies reporting essential oils, control or comparator information, doses, and outcomes against major honeybee diseases, reorganized by disease group.

Disease/Target	Oil or Compound	Dose/Concentration	Model/Control or Comparator	Main Outcome	Critical Scientific Interpretation	Reference
**Bacterial brood diseases: American foulbrood and European foulbrood**						
AFB/*P. larvae*	Cinnamon; camphor; citronellal; bay; clove; oregano; thymol; α-terpinene	10–10,000 µg/mL (reported as 10–10,000 ppm; oil-dependent)	In vitro growth inhibition (72 h); growth-control comparison, no larval or colony comparator	Vegetative growth inhibition	Useful antibacterial screening only; it does not support AFB control because endospore viability, infected-larva outcomes, and hive-matrix contamination were not assessed.	[57]
AFB/*P. larvae*	Thyme oil + cinnamon oil + thymol	62.5% + 12.5% + 25% mixture	In vitro bacterial inhibition; untreated growth-control comparator, no endospore or colony comparator	Synergistic vegetative-cell inhibition	Synergy suggests formulation potential, but the endpoint remains vegetative growth; no evidence is provided for endospore inactivation, recurrence prevention, or colony-level control.	[59]
AFB/infected nuclei	*Cinnamomum* sp. oil	400 mg/kg (semi-solid feed)	Apiary nuclei (autumn–winter); control/comparator strength is limited in the summarized field design	Reduced clinical signs	Clinical improvement in nuclei is encouraging, but the seasonal small-unit field design, limited control information, and unresolved spore reservoir restrict inference.	[63]
AFB/infected colonies	*Cinnamomum zeylanicum* oil	Two doses of 1000 µg/mL per hive	Field trial; untreated infected colony control	Reduced infected larvae	Stronger field evidence because infected controls were included, but endospore-reservoir clearance, recurrence, residues, and safety endpoints remain unproven.	[60]
EFB-associated bacteria	Cinnamon oil; thymol	10 µg/mL (reported as 10 ppm; 72 h)	In vitro P. alvei growth inhibition; growth-control comparison, no *M. plutonius* larval EFB model	Complete vegetative growth inhibition	Activity against P. alvei, a secondary invader, does not establish EFB control against *M. plutonius*-infected larvae or colony-level disease.	[57]
AFB/EFB bacteria	Oregano; juniper; sage; thyme; cinnamon; cumin; clove; black pepper	Spot-on-agar and resazurin microdilution screening	In vitro pathogen screening plus adult-worker vapor exposure; laboratory controls, no larval or colony comparator	Broad antibacterial activity against vegetative cells; toxicity increased with longer vapor exposure	Translational relevance improves by pairing pathogen inhibition with adult-worker safety, but adult vapor tolerance cannot substitute for larval, brood, queen, endospore, and field controls.	[66]
**Varroosis: laboratory mite toxicity, selectivity, and delivery-dependent field efficacy**						
Varroosis/*V. destructor*	*Tagetes minuta* oil	LD_50_ ≈ 4.37 mg/cage (24 h; complete-exposure assay)	Laboratory complete-exposure assay; mortality control, no hive-level comparator	Acaricidal activity	The LD_50_ endpoint supports mite toxicity, but cage exposure does not predict release kinetics, brood exposure, residues, or colony-level efficacy.	[68]
Varroosis/*V. destructor*	*Schinus molle* oil	LD_50_ ≈ 2.65 µL/cage (24 h; complete-exposure assay)	Laboratory complete-exposure assay; mite–bee selectivity comparison, no hive-level comparator	High mite mortality	High selectivity is promising, but laboratory selectivity can overestimate field safety; delivery, repellency, brood exposure, and colony performance remain untested.	[69]
Varroosis/*V. destructor*	Anethole; *Cymbopogon citratus* oil	LC_50_ ≈ 304.9 µg/mL (anethole) and 474.13 µg/mL (Cymbopogon citratus oil)	Laboratory mite and bee toxicity assay; acute selectivity comparator, no colony-level comparator	High mite toxicity; low bee toxicity	Bee-selectivity reporting strengthens the model, but acute laboratory endpoints do not address chronic exposure, brood safety, residues, or formulation performance.	[71]
Varroosis/*V. destructor*	*Origanum heracleoticum* oil	1000–2000 µg/mL (reported as 1–2 mg/mL; fumigation/contact)	Laboratory fumigation/contact assays; laboratory mortality controls, no field comparator	80–90.9% mortality	High mite mortality is model-dependent; without field delivery controls, the result cannot define practical Varroa control.	[73]
Varroosis/*V. destructor*	Thymol; thymol + carvacrol	71 µg/mL; 42 µg/mL (4 h)	Laboratory short-exposure assay; single-compound and combination comparator, no colony control	High acaricidal activity	Combination treatment may increase potency, but short-exposure LC_50_ data do not establish sustained release, selectivity, brood safety, or colony tolerance.	[78]
Varroosis/colonies	Oregano oil	Pads vs. continuous-release vaporizers	Field trial; delivery-system comparator (impregnated pads vs. continuous-release vaporizers)	57.8% vs. 97.4% efficacy	Direct delivery comparison is valuable and shows that formulation drives efficacy; generalization requires replicated apiaries, seasonal controls, and colony-strength endpoints.	[87]
**Nosemosis: field outcomes with unresolved stage-specific mechanisms**						
Nosemosis/*Nosema* spp.	Thyme; peppermint; eucalyptus oils	Field treatment	Naturally infected colonies; oil-treatment comparison, control/reference reporting should be explicit	84%, 77.45%, 76.10% efficacy	Field percentages are useful but insufficient mechanistically; efficacy should be linked to untreated/reference controls, baseline infection, polar-tube or intracellular endpoints, and durable colony recovery.	[95]
**Chalkbrood: in vitro antifungal screening and limited colony-level evidence**						
Chalkbrood/*A. apis*	Camphor; citronellal; cinnamon oils	100 µg/mL (reported as 100 ppm; 72–168 h)	In vitro fungal inhibition; growth-control comparison, no larval or brood model	Fungal growth inhibition	This is early antifungal screening only; agar inhibition does not demonstrate brood protection, recurrence control, or field-level chalkbrood reduction.	[57]
Chalkbrood/*A. apis*	Coriander; basil; *Tagetes minuta* oils	0.7–0.9 µL/mL (reported as 700–900 µL/L)	In vitro fungistatic assay; growth-control comparison, no brood or field comparator	Fungistatic activity	Fungistatic activity is oil- and dose-specific; no evidence shows that effective exposure can be achieved in infected brood under colony conditions.	[96]
Chalkbrood/colonies	Cedar oil; thymol	4% cedar oil; thymol treatment	Field trial; treatment comparison (cedar oil vs. thymol), untreated/reference and recurrence controls require clearer reporting	100% vs. 63.22–96.94% reduction	The colony-level outcome is valuable, but interpretation depends on control design, recurrence monitoring, colony strength, brood microclimate, and seasonal replication.	[101]

Note: Studies are grouped by disease category and arranged from laboratory screening toward field-level evidence where available. Control or comparator information is summarized in the Model/Control or Comparator column; where control structures are unclear or weak, this is treated as a limitation rather than assumed evidence of efficacy. Concentration units are standardized as µg/mL for ppm and mg/mL values and as µL/mL for µL/L values. LD_50_ values in mg/cage or µL/cage and feed-, hive-, or formulation-level doses are retained as exposure-unit endpoints.

**Table 5:** Evidence level and critical appraisal of essential-oil-based approaches for major honeybee diseases.

Disease	Predominant Evidence Level	Relative Strength of Evidence	Main Evidence Pattern	Key Limitations and Bias Risks
AFB	In vitro studies with limited semi-field and field observations	Strong for inhibition of *P. larvae* vegetative cells; no demonstrated sporicidal activity; weak to moderate for colony-level control	Cinnamon, thyme, clove, oregano, tea tree, thymol, eugenol, carvacrol, and cinnamaldehyde show antibacterial activity; some colony studies report reduced clinical signs. Recent laboratory data now include parallel screening against *P. larvae* and *M. plutonius* with adult worker exposure endpoints.	Spore persistence, brood delivery, recurrence, treatment timing, limited replication, and contradictory field outcomes reduce practical certainty. Unsuccessful field applications and insufficient lavender-oil efficacy indicate that positive in vitro data may not translate into disease prevention.
EFB	Mainly in vitro studies; few disease-specific colony trials	Preliminary to moderate	Selected oils inhibit EFB-associated bacteria, but direct evidence against *M. plutonius* and full disease expression is comparatively sparse.	Use of surrogate or secondary bacterial targets, multifactorial pathogenesis, larval nutrition, colony stress, and scarce field validation limit inference. Absence of robust colony-level EFB trials prevents conclusions about recurrence prevention or practical disease reversal.
Varroosis	Laboratory, semi-field, and field studies	Strongest for thymol-based formulations; variable for other oils	Many oils are acaricidal in laboratory assays; formulation and release system strongly determine colony-level efficacy. Recent nanoemulsion, semi-field, and integrated pest-management-oriented field studies strengthen formulation-level evidence.	Dose units, mite source, exposure route, temperature, brood status, bee selectivity, and reporting of controls differ widely among studies. Newer work also shows that carrier systems, brood safety, residues, repeated application, and colony strength can determine practical suitability. Moderate field efficacy, statistically unclear nanoemulsion effects, and possible colony-strength penalties limit interpretation of recent applied studies.
Nosemosis	Cage/laboratory assays and limited field studies	Moderate but heterogeneous	Thymol and several plant-derived products show outcome-level reductions in spore load or improved survival in selected models; however, the affected microsporidian stage remains unresolved.	Spore-load reduction may not predict colony recovery; infection level, *Nosema* species, feeding route, gut effects, and endpoints vary across studies. Positive spore-load outcomes may overstate clinical value when colony recovery and microbiota effects are not assessed. Stage-resolved assays of polar tube extrusion, intracellular parasite burden, histopathology, epithelial renewal, and microbiota effects are largely missing.
Chalkbrood	Predominantly in vitro; limited colony-level studies	Moderate for antifungal activity; limited for reproducible field control	Several oils inhibit A. apis growth or spore development; cedar oil and thymol have been linked with reduced chalkbrood mummies in field observations.	Disease expression depends on brood temperature, humidity, colony strength, hygienic behavior, and season; field replication remains limited. Single-context field success may not predict performance across seasons, brood conditions, or apiary environments.

**Table 6:** Main limitations and recommended research priorities for essential-oil-based honeybee disease control.

Limitation	Scientific Problem	Recommended Priority
Chemical variability	Oils derived from the same plant species may differ substantially in their active constituents	Report botanical origin, extraction method, and detailed chemical composition
Laboratory–field gap	In vitro efficacy does not reliably predict colony-level performance	Conduct semi-field and field trials using standardized endpoints
Bee safety	Adult-worker mortality alone is insufficient for estimating colony tolerance to essential-oil exposure because sublethal toxicity and microbiome disruption may occur without immediate death	Assess acute, chronic, contact, fumigation, ingestion, and residue-mediated exposure across relevant colony compartments together with microbiome-level safety endpoints
Queen and brood safety	Adult-worker tolerance does not guarantee queen survival, oviposition, retinue stability, supersedure risk, larval survival, pupal emergence, or brood-pattern integrity	Include queen mortality, egg laying, retinue behavior, supersedure, worker acceptance, larval and pupal survival, brood-removal behavior, and brood-pattern scoring
Sublethal behavior and physiology	Essential-oil vapors may alter fanning, avoidance, hygienic behavior, learning, olfactory responsiveness, gene expression, detoxification or oxidative-stress markers, and genotoxicity without causing immediate death	Combine mortality endpoints with behavioral assays, enzyme and biomarker testing, genotoxicity screening, and chronic low-dose exposure studies
Microbiota and chemical communication	Ingested oils may affect gut bacterial balance and potentially suppress beneficial symbionts that contribute to pathogen resistance, while volatile odorants may mask or modify responses to queen, brood, alarm, and orientation pheromones	Include microbiota profiling, feeding and digestion endpoints, quantification of core symbionts, pathogen-challenge or secondary-infection endpoints, and assays for queen and brood pheromone responsiveness, alarm signaling, Nasonov-related orientation, and colony-level social regulation
Formulation	Volatility and chemical instability affect release rate and biological efficacy	Develop controlled-release systems and standardized delivery methods Validate nanoemulsions, microencapsulation, and controlled-release carriers under realistic brood, temperature, and colony-strength conditions. Assess carrier scalability, environmental fate, and regulatory acceptance before practical recommendation.
Evidence interpretation	Screening studies may overrepresent positive findings and lack comparability	Compare candidates with established controls and report negative as well as positive results
Negative and inconsistent outcomes	Positive screening results may dominate the literature, while unsuccessful field applications, weak selectivity, or non-significant effects are less visible	Report ineffective doses, field failures, toxicity thresholds, and non-significant results alongside successful outcomes
Commercial translation	Volatility, variable composition, storage stability, residue concerns, regulatory approval, and product standardization may prevent practical adoption	Evaluate shelf life, batch reproducibility, residue behavior, cost, user safety, and regulatory feasibility before recommending routine use

## Data Availability

No new data were created or analyzed in this study.

## References

[1] Morse R.A., Calderone N.W. (2000). The value of honey bees as pollinators of US crops in 2000. Bee Cult..

[2] Turo K.J., Reilly J.R., Fijen T.P.M., Magrach A., Winfree R. (2024). Insufficient pollinator visitation often limits yield in crop systems worldwide. Nat. Ecol. Evol..

[3] Neumann P., Carreck N.L. (2010). Honey bee colony losses. J. Apic. Res..

[4] Mutinelli F., Pinto A., Barzon L., Toson M. (2022). Some considerations about winter colony losses in Italy according to the COLOSS questionnaire. Insects.

[5] Potts S.G., Biesmeijer J.C., Kremen C., Neumann P., Schweiger O., Kunin W.E. (2010). Global pollinator declines: Trends, impacts and drivers. Trends Ecol. Evol..

[6] Evans J.D., Schwarz R.S. (2011). Bees brought to their knees: Microbes affecting honey bee health. Trends Microbiol..

[7] Genersch E., Forsgren E., Pentikäinen J., Ashiralieva A., Rauch S., Kilwinski J., Fries I. (2006). Reclassification of *Paenibacillus larvae* subsp. *pulvifaciens* and *Paenibacillus larvae* subsp. *larvae* as *Paenibacillus larvae* without subspecies differentiation. Int. J. Syst. Evol. Microbiol..

[8] Hansen H., Brødsgaard C.J. (1999). American foulbrood: A review of its biology, diagnosis and control. Bee World.

[9] Genersch E. (2010). American foulbrood in honeybees and its causative agent, *Paenibacillus larvae*. J. Invertebr. Pathol..

[10] Matović K., Žarković A., Debeljak Z., Vidanović D., Vasković N., Tešović B., Ćirić J. (2023). American Foulbrood—Old and Always New Challenge. Vet. Sci..

[11] Wilkins S., Brown M., Cuthbertson A.G.S. (2007). The incidence of honey bee pests and diseases in England and Wales. Pest Manag. Sci..

[12] Roetschi A., Berthoud H., Kuhn R., Imdorf A. (2008). Infection rate based on quantitative real-time PCR of *Melissococcus plutonius*, the causal agent of European foulbrood, in honeybee colonies before and after apiary sanitation. Apidologie.

[13] Budge G.E., Burns N., Takamatsu D., Erler S., Forsgren E., Grossar D., Hornitzky M., Milbrath M., Pufal H., Tomkies V. (2025). Standard methods for European foulbrood research 2.0. J. Apic. Res..

[14] Rosenkranz P., Aumeier P., Ziegelmann B. (2010). Biology and control of *Varroa destructor*. J. Invertebr. Pathol..

[15] Nazzi F., Le Conte Y. (2016). Ecology of *Varroa destructor*, the major ectoparasite of the western honey bee, *Apis mellifera*. Annu. Rev. Entomol..

[16] Warner S., Pokhrel L.R., Akula S.M., Ubah C.S., Richards S.L., Jensen H., Kearney G.D. (2024). A scoping review on the effects of *Varroa* mite (*Varroa destructor*) on global honey bee decline. Sci. Total Environ..

[17] de Miranda J.R., Genersch E. (2010). Deformed wing virus. J. Invertebr. Pathol..

[18] Martin S.J., Highfield A.C., Brettell L., Villalobos E.M., Budge G.E., Powell M., Nikaido S., Schroeder D.C. (2012). Global honey bee viral landscape altered by a parasitic mite. Science.

[19] de Miranda J.R., Cordoni G., Budge G. (2010). The Acute bee paralysis virus–Kashmir bee virus–Israeli acute paralysis virus complex. J. Invertebr. Pathol..

[20] Ribière M., Olivier V., Blanchard P. (2010). Chronic bee paralysis: A disease and a virus like no other? *J*. Invertebr. Pathol..

[21] Budge G.E., Simcock N.K., Holder P.J., Shirley M.D.F., Brown M.A., Van Weymers P.S.M., Evans D.J., Rushton S.P. (2020). Chronic bee paralysis as a serious emerging threat to honey bees. Nat. Commun..

[22] Paxton R.J. (2010). Does infection by *Nosema ceranae* cause “Colony Collapse Disorder” in honey bees (*Apis mellifera*)?. J. Apic. Res..

[23] Kurze C., Le Conte Y., Kryger P., Lewkowski O., Müller T., Moritz R.F.A. (2018). Infection dynamics of *Nosema ceranae* in honey bee midgut and host cell apoptosis. J. Invertebr. Pathol..

[24] Aronstein K.A., Murray K.D. (2010). Chalkbrood disease in honey bees. J. Invertebr. Pathol..

[25] Mutinelli F. (2003). European legislation governing the authorization of veterinary medicinal products with particular reference to the use of drugs for the control of honey bee diseases. Apiacta.

[26] Lodesani M., Costa M. (2005). Limits of chemotherapy in beekeeping: Development of resistance and the problem of residues. Bee World.

[27] Milani N. (1995). The resistance of *Varroa jacobsoni* Oud. to pyrethroids: A laboratory assay. Apidologie.

[28] Mullin C.A., Frazier M., Frazier J.L., Ashcraft S., Simonds R., vanEngelsdorp D., Pettis J.S. (2010). High levels of miticides and agrochemicals in North American apiaries: Implications for honey bee health. PLoS ONE.

[29] Huang W.F., Solter L.F., Yau P.M., Imai B.S. (2013). *Nosema ceranae* escapes fumagillin control in honey bees. PLoS Pathog..

[30] Hornitzky M. (2001). Literature Review of Chalkbrood: A Report for the RIRDC.

[31] Association Française de Normalisation (2000). Huiles Essentielles, Tome 2, Monographies Relatives aux Huiles Essentielles.

[32] Schmidt E., Baser K.H.C., Buchbauer G. (2010). Production of essential oils. Handbook of Essential Oils: Science, Technology, and Applications.

[33] Sell C., Baser K.H.C., Buchbauer G. (2010). Chemistry of essential oils. Handbook of Essential Oils: Science, Technology, and Applications.

[34] Dhifi W., Bellili S., Jazi S., Bahloul N., Mnif W. (2016). Essential oils’ chemical characterization and investigation of some biological activities: A critical review. Medicines.

[35] Chouhan S., Sharma K., Guleria S. (2017). Antimicrobial activity of some essential oils—Present status and future perspectives. Medicines.

[36] Imdorf A., Bogdanov S., Ochoa R.I., Calderone N.W. (1999). Use of essential oils for the control of *Varroa jacobsoni* Oud. in honey bee colonies. Apidologie.

[37] Sangwan N.S., Farooqi A.H.A., Shabih F., Sangwan R.S. (2001). Regulation of essential oil production in plants. Plant Growth Regul..

[38] Vogt T. (2010). Phenylpropanoid biosynthesis. Mol. Plant.

[39] Gupta I., Singh R., Muthusamy S., Sharma M., Grewal K., Singh H.P., Batish D.R. (2023). Plant Essential Oils as Biopesticides: Applications, Mechanisms, Innovations, and Constraints. Plants.

[40] Burt S. (2004). Essential oils: Their antibacterial properties and potential applications in foods—A review. Int. J. Food Microbiol..

[41] Pellegrini M.C., Alonso-Salces R.M., Umpierrez M.L., Rossini C., Fuselli S.R. (2017). Chemical composition, antimicrobial activity, and mode of action of essential oils against *Paenibacillus larvae*, etiological agent of American foulbrood on *Apis mellifera*. Chem. Biodivers..

[42] Wani A.R., Yadav K., Khursheed A., Rather M.A. (2021). An updated and comprehensive review of the antiviral potential of essential oils and their chemical constituents with special focus on their mechanism of action against various influenza and coronaviruses. Microb. Pathog..

[43] Palmer-Young E.C., Tozkar C.Ö., Schwarz R.S., Chen Y., Irwin R.E., Adler L.S., Evans J.D. (2017). Nectar and pollen phytochemicals stimulate honey bee (Hymenoptera: Apidae) immunity to viral infection. J. Econ. Entomol..

[44] Parekh F., Daughenbaugh K.F., Flenniken M.L. (2021). Chemical stimulants and stressors impact the outcome of virus infection and immune gene expression in honey bees (*Apis mellifera*). Front. Immunol..

[45] Hsieh E.M., Berenbaum M.R., Dolezal A.G. (2020). Ameliorative effects of phytochemical ingestion on viral infection in honey bees. Insects.

[46] Boncristiani D.L., Tauber J.P., Palmer-Young E.C., Cao L., Collins W., Grubbs K., Lopez J.A., Meinhardt L.W., Nguyen V., Oh S. (2021). Impacts of diverse natural products on honey bee viral loads and health. Appl. Sci..

[47] Tian J., Ban X., Zeng H., He J., Chen Y., Wang Y. (2012). The mechanism of antifungal action of essential oil from dill (*Anethum graveolens* L.) on *Aspergillus flavus*. PLoS ONE.

[48] Isman M.B. (2000). Plant essential oils for pest and disease management. Crop Prot..

[49] Ryan M.F., Byrne O. (1988). Plant-insect coevolution and inhibition of acetylcholinesterase. J. Chem. Ecol..

[50] Enan E. (2001). Insecticidal activity of essential oils: Octopaminergic sites of action. Comp. Biochem. Physiol. C Toxicol. Pharmacol..

[51] Priestley C.M., Williamson E.M., Wafford K.A., Sattelle D.B. (2003). Thymol, a constituent of thyme essential oil, is a positive allosteric modulator of human GABAA_AA receptors and a homo-oligomeric GABA receptor from *Drosophila melanogaster*. Br. J. Pharmacol..

[52] Amorati R., Valgimigli L. (2018). Methods to measure the antioxidant activity of phytochemicals and plant extracts. J. Agric. Food Chem..

[53] Foti M.C., Ingold K.U. (2003). Mechanism of inhibition of lipid peroxidation by γ-terpinene, an unusual and potentially useful hydrocarbon antioxidant. J. Agric. Food Chem..

[54] Pătruică S., Moț D., Popovici D. (2017). The effect of using medicinal plant extracts upon the health of bee colonies. Rom. Biotechnol. Lett..

[55] Ewert A.M., Simone-Finstrom M., Read Q., Husseneder C., Ricigliano V. (2023). Effects of ingested essential oils and propolis extracts on honey bee (Hymenoptera: Apidae) health and gut microbiota. J. Insect Sci..

[56] Koev K., Ganeva M., Orozova P. (2026). Natural Products—Part of a Strategy to Mitigate the Impact of Climate Change on Honey Bees. Agriculture.

[57] Calderone N.W., Shimanuki H., Allen-Wardell G. (1994). An in vitro evaluation of botanical compounds for the control of the honey bee pathogens *Bacillus larvae* and *Ascosphaera apis*, and the secondary invader *B. alvei*. J. Essent. Oil Res..

[58] Alippi A.M., Ringuelet J.A., Cerimele E.L., Re M.S., Henning C.P. (1996). Antimicrobial activity of some essential oils against *Paenibacillus larvae*, the causal agent of American foulbrood disease. J. Herbs Spices Med. Plants.

[59] Fuselli S.R., García de la Rosa S.B., Gende L.B., Eguaras M.J., Fritz R. (2006). Inhibition of *Paenibacillus larvae* employing a mixture of essential oils and thymol. Rev. Argent. Microbiol..

[60] Gende L.B., Maggi M.D., Damiani N., Fritz R., Eguaras M.J., Floris I. (2009). Advances in the apiary control of the honeybee American foulbrood with cinnamon (*Cinnamomum zeylanicum*) essential oil. Bull. Insectology.

[61] Santos R.C.V., Lopes L.Q.S., Alves C.F.S., Fausto V.P., Pizzutti K., Barboza V., de Souza M.E., Raffin R.P., Gomes P., Takamatsu D. (2014). Antimicrobial activity of tea tree oil nanoparticles against American and European foulbrood disease agents. J. Asia-Pac. Entomol..

[62] Ansari M.J., Al-Ghamdi A., Usmani S., Al-Waili N., Nuru A., Sharma D., Khan K.A., Kaur M., Omer M. (2016). In vitro evaluation of the effects of some plant essential oils on *Paenibacillus larvae*, the causative agent of American foulbrood. Biotechnol. Biotechnol. Equip..

[63] Floris I., Carta C., Moretti L. (1996). Activity of various essential oils against *Bacillus larvae* White in vitro and in apiary trials. Apidologie.

[64] Albo G.N., Henning C., Ringuelet J., Reynaldi F.J., De Giusti M.R., Alippi A.M. (2003). Evaluation of some essential oils for the control and prevention of American foulbrood disease in honey bees. Apidologie.

[65] Puvača N. (2022). Influence of lavender essential oil (*Lavandula angustifolia*) and oxytetracycline in nutrition of honey bees, prevention of American foulbrood and overall welfare. J. Hell. Vet. Med. Soc..

[66] Marianelli C., Narciso L. (2026). Effects of essential oils on foulbrood bacteria and honey bee workers (*Apis mellifera*) under laboratory conditions. Front. Insect Sci..

[67] Hassona N.M.K. (2017). Using natural products to control foulbrood diseases in honey bee *Apis mellifera* L. colonies under Egyptian conditions. Menoufia J. Plant Prot..

[68] Eguaras M.J., Fuselli S., Gende L., Fritz R., Ruffinengo S.R., Clemente G., Gonzalez A., Bailac P.N., Ponzi M.I. (2005). An in vitro evaluation of *Tagetes minuta* essential oil for the control of the honeybee pathogens *Paenibacillus larvae* and *Ascosphaera apis*, and the parasitic mite *Varroa destructor*. J. Essent. Oil Res..

[69] Ruffinengo S., Eguaras M., Floris I., Faverin C., Bailac P., Ponzi M. (2005). LD50 and repellent effects of essential oils from Argentinian wild plant species on *Varroa destructor*. J. Econ. Entomol..

[70] Ruffinengo S.R., Maggi M.D., Fuselli S., Fiorella G., Negri P., Brasesco C., Satta A., Floris I., Eguaras M.J. (2014). Bioactivity of microencapsulated essential oils and perspectives of their use in the control of *Varroa destructor*. Bull. Insectology.

[71] Sabahi Q., Hamiduzzaman M.M., Barajas-Pérez J.S., Tapia-Gonzalez J.M., Guzman-Novoa E. (2018). Toxicity of anethole and the essential oils of lemongrass and sweet marigold to the parasitic mite *Varroa destructor* and their selectivity for honey bee (*Apis mellifera*) workers and larvae. Psyche.

[72] Bava R., Castagna F., Piras C., Palma E., Cringoli G., Musolino V., Lupia C., Perri M.R., Statti G., Britti D. (2021). In vitro evaluation of acute toxicity of five *Citrus* spp. essential oils towards the parasitic mite *Varroa destructor*. Pathogens.

[73] Castagna F., Bava R., Piras C., Carresi C., Musolino V., Lupia C., Marrelli M., Conforti F., Palma E., Britti D. (2022). Green veterinary pharmacology for honey bee welfare and health: *Origanum heracleoticum* L. (Lamiaceae) essential oil for the control of the *Apis mellifera* varroatosis. Vet. Sci..

[74] Erdoğan Y. (2026). Chemical composition acaricidal activity and honeybee safety of essential oils from *Origanum acutidens*, *Satureja hortensis*, and *Juniperus communis* against *Varroa destructor*. Ital. J. Anim. Sci..

[75] Manzo R.M., Iglesias A.E., Guajardo J.J., Amaturi C.A., Freeman B.D., López de Armentia J., Rizzuto S., Maggi M.D. (2025). Bioactivity of essential oils from Patagonian wild plants: Acaricidal and insecticidal effects on *Varroa destructor* and *Apis mellifera*. Plants.

[76] Lindberg C.M., Melathopoulos A.P., Winston M.L. (2000). Laboratory evaluation of miticides to control *Varroa jacobsoni* (Acari: Varroidae), a honey bee (Hymenoptera: Apidae) parasite. J. Econ. Entomol..

[77] Gashout H.A., Guzmán-Novoa E. (2009). Acute toxicity of essential oils and other natural compounds to the parasitic mite *Varroa destructor* and to larval and adult worker honey bees (*Apis mellifera* L.). J. Apic. Res..

[78] Begna T., Ulziibayar D., Bisrat D., Jung C. (2023). Acaricidal toxicity of four essential oils, their predominant constituents, their mixtures against *Varroa* mite, and their selectivity to honey bees (*Apis cerana* and *A. mellifera)*. Insects.

[79] Bava R., Palma E., Bulotta R.M., Ruga S., Liguori G., Lombardi R., Lupia C., Marrelli M., Statti G., Musella V. (2025). Green veterinary pharmacology applied to beekeeping: Semi-field and field tests against *Varroa destructor*, using essential oil of bergamot (*Citrus bergamia*) and lemon (*Citrus limon*). Vet. Sci..

[80] Güneşdoğdu M. (2026). Colony-level efficacy of *Mentha piperita*, *Thymus vulgaris* and *Eucalyptus globulus* essential oil nanoemulsions against *Varroa destructor*. Exp. Appl. Acarol..

[81] Gamal Eldin N.K., Ebeid A.A., Sallam A.M., Basuny N.K., Elaidy W.K. (2024). Efficacy of thymol nanoemulsion against *Varroa destructor* mites infesting *Apis mellifera* colonies under the stress of abiotic factors in Egypt. Egypt. J. Basic Appl. Sci..

[82] Aurell D., Wall C., Bruckner S., Williams G.R. (2024). Combined treatment with amitraz and thymol to manage *Varroa destructor* mites (Acari: Varroidae) in *Apis mellifera* honey bee colonies (Hymenoptera: Apidae). J. Insect Sci..

[83] Damiani N., Gende L.B., Bailac P., Marcangeli J.A., Eguaras M.J. (2009). Acaricidal and insecticidal activity of essential oils on *Varroa destructor* (Acari: Varroidae) and *Apis mellifera* (Hymenoptera: Apidae). Parasitol. Res..

[84] Ghasemi V., Moharramipour S., Tahmasbi G.H. (2016). Laboratory cage studies on the efficacy of some medicinal plant essential oils for controlling varroosis in *Apis mellifera* (Hym.: Apidae). Syst. Appl. Acarol..

[85] Calderone N.W., Spivak M. (1995). Plant extracts for control of the parasitic mite *Varroa jacobsoni* (Acari: Varroidae) in colonies of the western honey bee (Hymenoptera: Apidae). J. Econ. Entomol..

[86] Melathopoulos A.P., Pernal S.F., Moller E., Baumgartner W., Guzmán-Novoa E. (2010). A spring evaluation of thymol formulated in a sucrose dust for the control of *Varroa destructor*, a parasite of the honey bee (*Apis mellifera*) in Alberta, Canada. Sci. Bee Cult..

[87] Sabahi Q., Gashout H., Kelly P.G., Guzman-Novoa E. (2017). Continuous release of oregano oil effectively and safely controls *Varroa destructor* infestations in honey bee colonies in a northern climate. Exp. Appl. Acarol..

[88] Conti B., Bocchino R., Cosci F., Ascrizzi R., Flamini G., Bedini S. (2020). Essential oils against *Varroa destructor*: A soft way to fight the parasitic mite of *Apis mellifera*. J. Apic. Res..

[89] Dussaubat C., Brunet J.-L., Higes M., Colbourne J.K., Lopez J., Choi J.-H., Martin-Hernández R., Botías C., Cousin M., McDonnell C. (2012). Gut pathology and responses to the microsporidium *Nosema ceranae* in the honey bee *Apis mellifera*. PLoS ONE.

[90] Panek J., Paris L., Roriz D., Mone A., Dubuffet A., Delbac F., Diogon M., El Alaoui H. (2018). Impact of the microsporidian *Nosema ceranae* on the gut epithelium renewal of the honeybee, *Apis mellifera*. J. Invertebr. Pathol..

[91] Maistrello L., Lodesani M., Costa C., Leonardi F., Marani G., Caldon M., Mutinelli F., Granato A. (2008). Screening of natural compounds for the control of nosema disease in honeybees (*Apis mellifera*). Apidologie.

[92] Costa C., Lodesani M., Maistrello L. (2010). Effect of thymol and resveratrol administered with candy or syrup on the development of *Nosema ceranae* and on the longevity of honeybees (*Apis mellifera* L.) in laboratory conditions. Apidologie.

[93] Glavinić U., Blagojević J., Ristanić M., Stevanović J., Lakić N., Mirilović M., Stanimirović Z. (2022). Use of thymol in *Nosema ceranae* control and health improvement of infected honey bees. Insects.

[94] Kim J.H., Park J., Lee J.K. (2016). Evaluation of antimicrosporidian activity of plant extracts on *Nosema ceranae*. J. Apic. Sci..

[95] Özüiçli M., Girişgin A.O., Diker A.İ., Baykalır Y., Kısadere İ., Aydın L. (2023). The efficacy of thyme, peppermint, eucalyptus essential oils, and nanoparticle ozone on nosemosis in honey bees. Kafkas Univ. Vet. Fak. Derg..

[96] Larrán S., Ringuelet J.A., Carranza M.R., Henning C.P., Ré M.S., Cerimele E.L., Urrutía M.I. (2001). In vitro fungistatic effect of essential oils against *Ascosphaera apis*. J. Essent. Oil Res..

[97] Kloucek P., Smid J., Flesar J., Havlik J., Titera D., Rada V., Drabek O., Kokoska L. (2012). In vitro inhibitory activity of essential oil vapors against *Ascosphaera apis*. Nat. Prod. Commun..

[98] Ansari M.J., Al-Ghamdi A., Usmani S., Khan K.A., Alqarni A.S., Kaur M., Al-Waili N. (2017). In vitro evaluation of the effects of some plant essential oils on *Ascosphaera apis*, the causative agent of chalkbrood disease. Saudi J. Biol. Sci..

[99] Nardoni S., D’Ascenzi C., Rocchigiani G., Papini R.A., Pistelli L., Formato G., Najar B., Mancianti F. (2018). Stonebrood and chalkbrood in *Apis mellifera* causing fungi: In vitro sensitivity to some essential oils. Nat. Prod. Res..

[100] Pusceddu M., Floris I., Mangia N.P., Angioni A., Satta A. (2021). In vitro activity of several essential oils extracted from aromatic plants against *Ascosphaera apis*. Vet. Sci..

[101] Mourad A.K., Zaghloul O.A., El Kady M.B., Nemat F.M., Morsy M.E. (2005). A novel approach for the management of the chalkbrood disease infesting honeybee *Apis mellifera* L. (Hymenoptera: Apidae) colonies in Egypt. Commun. Agric. Appl. Biol. Sci..

[102] Bonilla-Rosso G., Engel P. (2018). Functional roles and metabolic niches in the honey bee gut microbiota. Curr. Opin. Microbiol..

[103] Powell J.E., Carver Z., Leonard S.P., Moran N.A. (2021). Field-realistic tylosin exposure impacts honey bee microbiota and pathogen susceptibility, which is ameliorated by native gut probiotics. Microbiol. Spectr..

[104] Caren J., Zhu Y.C., Read Q.D., Du Y. (2025). Risk assessment of effects of essential oils on honey bees (*Apis mellifera* L.). Insects.

[105] Jin W., Chen H., Wang Z., An H. (2026). Recent progress in the construction and application of controlled-release essential oil formulations. Pestic. Biochem. Physiol..

[106] Charpentier G., Vidau C., Ferdy J.B., Tabart J., Vétillard A. (2014). Lethal and sub-lethal effects of thymol on honeybee (*Apis mellifera*) larvae reared in vitro. Pest Manag. Sci..

[107] Floris I., Satta A., Cabras P., Garau V.L., Angioni A. (2004). Comparison between two thymol formulations in the control of *Varroa destructor*: Effectiveness, persistence, and residues. J. Econ. Entomol..

[108] Whittington R., Winston M.L., Melathopoulos A.P., Higo H.A. (2000). Evaluation of the botanical oils neem, thymol, and canola sprayed to control *Varroa jacobsoni* Oud. (Acari: Varroidae) and *Acarapis woodi* (Acari: Tarsonemidae) in colonies of honey bees (*Apis mellifera* L., Hymenoptera: Apidae). Am. Bee J..

[109] Glavinić U., Rajković M., Ristanić M., Stevanović J., Vejnović B., Djelić N., Stanimirović Z. (2023). Genotoxic potential of thymol on honey bee DNA in the comet assay. Insects.

[110] Mondet F., Goodwin M., Mercer A. (2011). Age-related changes in the behavioural response of honeybees to Apiguard, a thymol-based treatment used to control the mite *Varroa destructor*. J. Comp. Physiol. A Neuroethol. Sens. Neural Behav. Physiol..

[111] Bonnafé E., Drouard F., Hotier L., Carayon J.-L., Marty P., Treilhou M., Armengaud C. (2015). Effect of a thymol application on olfactory memory and gene expression levels in the brain of the honeybee *Apis mellifera*. Environ. Sci. Pollut. Res..

[112] Chapuy C., Ribbens L., Renou M., Dacher M., Armengaud C. (2019). Thymol affects congruency between olfactory and gustatory stimuli in bees. Sci. Rep..

[113] Colin T., Lim M.Y., Quarrell S.R., Allen G.R., Barron A.B. (2019). Effects of thymol on European honey bee hygienic behaviour. Apidologie.

[114] Gryboś A., Staniszewska P., Bryś M.S., Strachecka A. (2025). The pheromone landscape of *Apis mellifera*: Caste-determined chemical signals and their influence on social dynamics. Molecules.

